# Could clinical experience during clerkship enhance students’ clinical performance?

**DOI:** 10.1186/1472-6920-14-209

**Published:** 2014-10-02

**Authors:** Ji Young Kim, Sun Jung Myung

**Affiliations:** Office of Medical Education, Seoul National University College of Medicine, 103 Daehak-ro, Jongno-gu, Seoul, 110-799 Republic of Korea

**Keywords:** Objective structured clinical examination, Clinical clerkship, Medical education, Clinical competency

## Abstract

**Background:**

Medical students learn and practice various clinical skills during clinical clerkship. Patient encounters are important for developing clinical thinking, communication skills, and professional attitude. We investigated whether the amount of clinical experience during clerkship correlated with students’ clinical competency and students’ perception of effectiveness of their clerkship on it.

**Methods:**

Fourth year medical students undertook the Objective Structured Clinical Examinations (OSCE) in August 2012. Students provided the number of patients for whom they took medical histories or performed physical examinations during clerkship and provided feedback as to whether or not the clinical clerkship was helpful in preparing OSCE. The correlation between the OSCE score and number of patients was analyzed.

**Results:**

One hundred thirty students completed the questionnaire (86.6%). OSCE scores correlated with the total number of patients encountered for physical examinations (correlation coefficient, 0.274; *p* = 0.0105). Cumulative 3-year GPAs were positively correlated with OSCE scores (correlation coefficient, 0.330; *p* = 0.0001). Most (92.3%) answered that their clinical clerkship was helpful in preparing them for the OSCE; however, only 20% felt that their clinical clerkship was most helpful. Others felt that role playing (38.46%) or the guide book (33.84%) was most helpful.

**Conclusions:**

The amount of clinical experience during the students’ clerkship had a small but positive relationship with students’ clinical performance. Further research to elucidate the influence of clinical experience on clinical competency is needed.

**Electronic supplementary material:**

The online version of this article (doi:10.1186/1472-6920-14-209) contains supplementary material, which is available to authorized users.

## Background

Traditionally, we believe that clinical competence is developed from clinical experience. Learning clinical skills is a time-consuming and gradual process, and clerkship is the time in which students must learn and practice various clinical skills. During clinical clerkship, students encounter patients in the ward or at outpatient clinics, and these encounters are at the core of the program for developing students’ clinical competency [1, 2].

Nowadays, early clinical exposure is warranted by experts in medical education [3–6], and we encourage students to actively participate in clinical practice. We believe that these experiences will enhance the students’ clinical competency. However, clinical experience during clinical clerkship varies among students, and the relationship between students’ clinical experience and their subsequent performance has always been recognized as complex [7].

Although much effort has been made to find correlations between the number or variety of patients seen during clerkships and clinical performance at the end of the clerkship, evidence regarding the educational value of patient encounters is still limited. The nature of the examinations given, such as the written knowledge-based examinations and the variety of quality of clinical experiences, were reasons for the observed lack of correlations [8–10]. Additionally, the quality of the clinical supervisor was important for developing clinical competence [11, 12]. Although the influence of clerkship experiences on clinical competence yielded contradictory results, medical students evaluated the exposure to real patients as very important [10]. Real-life contextual experience by clinical exposure added value in acquiring clinical competence, whereas paper-based case tutorials also provided students with clinical knowledge [13].

Because it is important that we understand how students benefit from clinical experience and what types of experience are most beneficial, we tried to determine whether the amount of clinical experience during clerkship in each department correlates with the clinical competency of the students and evaluated medical students’ perceptions of the effectiveness of their clerkships on their clinical performance examination.

## Methods

The current undergraduate curriculum of Seoul National University College of Medicine (SNUCM; Seoul, South Korea) includes two years of preclinical study followed by two years of clinical study. The third year course is run by the departments of internal medicine, surgery, pediatrics, psychiatry, obstetrics and gynecology etc. The fourth year course is composed of elective clerkships during which each student can choose his or her own clerkship schedule. During clinical clerkship, students learn key components of clinical practice by encountering patients, practicing clinical skills, and attending tutoring sessions. The students are required to maintain a logbook that lists of their experiences with patients and exposure to core problems in each department.

We used the Objective Structured Clinical Examination (OSCE) as a tool for assessing clinical performance because measuring medical students’ clinical competency using OSCEs has become increasingly widespread, and evidence of the validity of the test is mounting [14–17].

In August 2012, all fourth-year medical students at SNUCM participated in the OSCE, which consists of 8 stations presenting the following 8 clinical cases: syncope, abdominal pain, palpitation, alcohol abuse problems, hemoptysis, polyuria, and constipation. The OSCE was developed by the Seoul Kyeonggi CPX Consortium. Each case required 16 minutes to administer, with 1 min to introduce the next case, 10 min for the student-standardized patient encounter, and 5 min after the encounter for the student to report the most probable diagnosis. The student’s performance was evaluated by trained standardized patients (SPs) using a checklist [18, 19] (see Additional file 1 for the format of the scoring rubric of OSCE), and major evaluation components are overall assessment, history taking, physical examination, physician’s manner, patient education, and physician-patient interaction (PPI). Evaluating performance by trained SPs using checklist is regarded as reliable methods and this has been verified by many previous reports [16, 18, 19]. At the end of the exam, the students were asked to complete a questionnaire inquiring about the number of patients for whom they took a medical history and physical examination based on the logbook that each student kept during their clerkship. The questionnaire also has items for students’ perceptions of the effectiveness of the clerkship on their clinical performance examination (see Additional file 2 for items).

Grade point average (GPA), OSCE scores from the encounters with standardized patients (including history taking, physical examination, and patient-physician interaction), and the number of patients encountered during clerkship (especially those for whom students took a history or conducted a physical examination) were obtained and a correlation analysis was done. Statistical analyses were performed using SPSS software (version 19.0; SPSS Inc., Chicago, IL, USA). A *p*-value < 0.05 was considered statistically significant. The institutional review board provided study approval.

## Results

### Number of patients encountered by medical students during clinical clerkship

A total of 150 fourth-year students underwent the OSCE examinations in August 2012. Among them, 130 students (86.6%) completed the questionnaire. The number of patients that students examined during their clinical clerkship varied widely by department and individual student, ranging from 0 to 30. The total number of patients encountered during clerkship per year ranged from 5 to 85 (Table 1).

**Table 1:** **Average number of patients encounter during students’ clerkship**

		IM	GS	OBGY	Ped	Psy	OS	EM	Total
History Taking	Min	2	0	0	1	1	0	0	5
	Max	30	30	10	16	20	10	10	85
	Mean	13.9	8.6	3.1	4.9	5.7	1.9	2.6	40.5
	SD	5.5	5.5	2.7	2.7	3.0	2.0	1.7	14.9
Physical Exam	Min	0	0	0	0	0	0	0	0
	Max	30	20	10	16	12	20	10	85
	Mean	12.9	7.9	2.5	4.5	3.6	1.7	2.3	35.4
	SD	5.4	5.2	2.6	2.7	3.4	2.3	1.6	14.3

IM, internal medicine; GS, surgery; OBGY, obstetrics and gynecology; Ped, pediatrics; Psy, psychiatry; OS, orthopedics; EM, emergency medicine.

Min, minimum; Max, maximum; SD, standard deviation.

### Correlation between the number of patients who medical students encountered during clerkship and their OSCE score

As shown in Table 2, the total OSCE score was statistically significantly correlated with the total number of patients the students conducted a physical examination on during their third year clinical clerkship. OSCE scores for history taking, physical examination, or PPI partly correlated with the number of patients encountered during internal medicine, surgery, pediatrics, and psychiatry clerkships. GPA was also significantly correlated with OSCE scores for total, history taking, physical examination, and PPI (Table 2).

**Table 2:** **Pearson correlations between the number of patients encountered during clerkship and OSCE score, GPA**

		History taking	Physical examination	PPI	Total score
IM (Hx)	r	0.053	0.016	0.142	0.090
IM (P/E)	r	0.122	0.090	0.189	0.148
GS (Hx)	r	0.194	0.230	0.190	0.224
GS (P/E)	r	0.249	0.242	0.269	0.278
OBGY (Hx)	r	0.125	0.013	0.178	0.133
OBGY (P/E)	r	0.083	-0.030	0.161	0.102
Ped (Hx)	r	0.050	0.037	0.164	0.089
Ped (P/E)	r	0.085	0.076	0.195	0.132
Psychiatry (Hx)	r	0.033	0.049	0.109	0.071
Psychiatry (P/E)	r	0.147	0.172	0.308	0.245
OS (Hx)	r	0.105	-0.006	0.081	0.072
OS (P/E)	r	0.074	-0.118	0.041	-0.003
EM (Hx)	r	0.120	0.105	0.194	0.166
EM (P/E)	r	0.116	0.073	0.202	0.156
Total (Hx)	r	0.156	0.119	0.238*	0.198
	p	0.148	0.174	0.036	0.095
Total (P/E)	r	0.227*	0.160	0.336*	0.274*
	p	0.045	0.148	<.0001	0.0105
GPA	r	0.271*	0.314*	0.244*	0.330*
	p	0.001	0.0003	0.005	0.0001

IM, internal medicine; GS, surgery; OBGY, obstetrics and gynecology; Ped, pediatrics; OS, orthopedics; EM, emergency medicine; Hx, history taking; P/E, physical examination; PPI, physician-patient interaction.

r = correlation coefficient.

p = adjusted *p*-value by Hochberg’s step up method.

*A *p*-value < .05 was considered statistically significant.

### Was clinical clerkship helpful for enhancing the clinical competency of students?

Most students answered that their clinical clerkship was helpful and enhanced their OSCE scores (92.3% of students scored over 3 on a Likert scale score of 5). However, students also replied that role playing with friends (38.46%) or reading books dealing with OSCE (33.84%) were most helpful when preparing for the OSCE rather than clinical clerkship.

## Discussion

In this study, we show that the amount of clinical experience during clerkship has a small but positive relationship with students’ clinical performance. In the belief that seeing more patients and encountering more core problems will result in better clinical performance of the students, academic medical school managers try to provide students with more clinical encounters with patients earlier in their medical school training. Although this belief may seem self-evident at first glance, contemporary education theories and data from a limited number of studies in medical education provide little affirmation for this concept, [20, 21] and the goals of these earlier clinical encounters may vary. Better academic and clinical performances are certainly among the most important goals.

SNUCM also designed a clinical clerkship schedule to facilitate interactions between medical students and patients. However, the wide variation in the number of patient encounters during clerkship showed that clinical clerkship is not yet standardized, and we can assume that there is some qualitative variation in clinical clerkships as well as quantitative ones. In our study, we were astonished by data showing that the minimum number of patient encounters was zero in certain clerkships. This could be explained by students’ under reporting their experiences with patients. Previous studies reported variable accuracy in logbooks, which usually involved significant under-reporting but little over reporting [22–24]. That is to say, students do not seem to overestimate exposure to patients or core problems, but they may not report every exposure. In addition, some clerkships emphasize observation and training in special skills rather than patient encounters, and in some clerkships, such as obstetrics and gynecology, access to patients is limited due to the privacy of patients, while most other clerkships set tasks for encountering patients and keeping medical records. Thus, a fair number of students do not have adequate clinical exposure to problems and lack the opportunity to acquire the skills necessary for competency as a physician [25–28].

In this study, students thought that the clinical clerkship was helpful for enhancing OSCE scores, but also felt that role playing or reading books dealing with OSCE were more helpful than their clinical clerkship. Previous studies reported that medical students evaluated exposure to real patients as being very important [10], while performance on the OSCE examination was related to well-organized study methods, not to clinical experience [7]. Another study reported that prior academic performance, rather than preparatory studying time, is a better predictor of OSCE outcomes [29]. Our results also show that GPA was correlated with OSCE scores. Clinical performance is very complex in nature and requires various training and teaching methods, and a certain level of knowledge is crucial for appropriate clinical performance.

Our study suggests that clinical experience during clerkship is weakly associated with student’s clinical performance. Although only small correlations were present between them, the amount of clinical experience cannot be overlooked. We analyzed only the amount of clinical experience, without considering the variety of experiences or the quality of the supervision. Teaching quality and a supportive house staff were regarded as important elements for improving student performance during clerkships [10, 30]. All of these should be considered when designing a clinical clerkship program.

We also focused on the differences between the number of patients for whom a medical history was taken and physical examinations were performed. Students do not always do a physical examination on every patient they encounter. The explanation for this could be any of the following: students may be hesitant to do a physical examination as they are not confident in their skills to conduct the physical examination, and are afraid of asking patients to take off’ their clothes before doing the physical examination. Additionally, many physicians do not do physical examination thoroughly, and the importance of the physical examination has declined with the development of diagnostic aids. Therefore, students may have little chance of observing physicians doing physical examinations during clerkship.

Our study has several limitations. First, the OSCE examination, especially those using checklist rating systems, could be insufficient for accurately evaluating the clinical competency of students. Moreover, we tested only 8 cases, which may not be a sufficient number to evaluate clinical competency. Second, the number of patients that students encountered during clerkship could be inaccurate. The students recorded the number of patients for whom they took a medical history and performed a physical examination based on the logbook, but the accuracy of students’ completion of the logbook was not systematically examined. Bias could be introduced in the recording of information. Finally, our study analyzed the correlation between the amount of clinical experience and the clinical competency of the student. We did not consider the qualitative aspects of the clinical experiences. Qualitative evaluations of clinical experiences as well as quantitative ones could have a significant correlation with the clinical performance scores of students.

## Conclusions

In conclusion, there was small but positive relationship between clinical experience and students’ performance on the OSCE. Increasing the amount of clinical experience as well as the quality of this experience should be re-enforced during clinical clerkship.

### Ethical approval

This study was approved by the Institutional Review Board of Seoul National University College of Medicine. (IRB No. C-1202-040-397).

## Electronic supplementary material

Additional file 1: **The format of the scoring rubric of OSCE.** (DOCX 14 KB)

Additional file 2: **Questionnaire for student.** (DOCX 15 KB)
