# Supplementary material for: Could clinical experience during clerkship enhance students’ clinical performance?
Source: BMC Med Educ. 2014 Oct 2;14:209. doi: 10.1186/1472-6920-14-209 (PMC4190391; doi:10.1186/1472-6920-14-209)
Supplement: Supplementary file 1 — Additional file 1: The format of the scoring rubric of OSCE. (DOCX 14 KB) [file 12909_2014_1035_MOESM1_ESM.docx]

**Additional file 1.** The format of the scoring rubric of OSCE

Total OSCE score = History taking score (30–40%) + Physical examination score (20–40%) + Physician-patient interaction score (20–40%)

History taking score = Number of correct checklist/number of total checklist for history taking (usually 10 ~ 15 items) x (30–40/100)

The history taking skills were scored dichotomously as 0 or 1 (indicating incorrect or correct performance, respectively). (ex; The student asked me when my abdominal pain had started.)

Physical examination score = Number of correct checklist/number of total checklist for physical examination (usually 5 ~ 10 items) x (20–40/100)

The physical exam skills were scored on a three-point scale (0, 0.5, and 1; 0.5 for partial credit). (ex; The student auscultated my abdomen correctly)

Physician-patient interaction score = Number of correct checklist/number of total checklist for physician-patient interaction (usually 5 ~ 7 items) x (20–40/100)

The physician patient interaction skills were scored on a four-point scale (0,1,2,and 3; poor, fair, good and excellent, respectively) (ex; The student is a good listener (eye contact, attentive hearing, etc.)

The number of checklist and the proportion of scores of each category vary depending on case scenario of OSCE.
