# Supplementary material for: Could clinical experience during clerkship enhance students’ clinical performance?
Source: BMC Med Educ. 2014 Oct 2;14:209. doi: 10.1186/1472-6920-14-209 (PMC4190391; doi:10.1186/1472-6920-14-209)
Supplement: Supplementary file 2 — Additional file 2: Questionnaire for student. (DOCX 15 KB) [file 12909_2014_1035_MOESM2_ESM.docx]

**Additional file 2.** Questionnaire for student

1. Please add the number of patients for whom you took a medical history, and for whom you performed a physical examination based on the logbook during each clerkship

|  | Clerkship | Number of patient for whom you took a medical history | Number of patient for whom you performed a physical examination |
| --- | --- | --- | --- |
|  |  |  |  |
| 1 | Internal medicine |  |  |
| 2 | Surgery |  |  |
| 3 | Obsterics & Gynecology |  |  |
| 4 | Pediatrics |  |  |
| 5 | Psychiatry |  |  |
| 6 | Orthopedics |  |  |
| 7 | Emergency medicine |  |  |

2. Clinical clerkship was helpful for enhancing my clinical performance.

① ② ③ ④ ⑤

(1=strongly disagree; 5=strongly agree)

3. What was most helpful for preparing for the OSCE examination?

① clinical clerkship

② reading books dealing with OSCE

③ role playing

④ feedback from friends and tutors

⑤ others (please specify: )
